# Lung donation and SARS‐CoV‐2 transmission: Missed detection versus missed opportunity?

**DOI:** 10.1002/iid3.603

**Published:** 2022-03-10

**Authors:** Jan Van Slambrouck, Dirk Van Raemdonck, Joost Wauters, Robin Vos, Peter Mombaerts, Laurens J. Ceulemans

**Affiliations:** ^1^ Department of Thoracic Surgery University Hospitals Leuven Leuven Belgium; ^2^ Laboratory of Respiratory Diseases and Thoracic Surgery (BREATHE), Department of Chronic Diseases and Metabolism KU Leuven Leuven Belgium; ^3^ Medical Intensive Care Unit University Hospitals Leuven Leuven Belgium; ^4^ Laboratory for Clinical Infectious and Inflammatory Disorders, Department of Microbiology, Immunology and Transplantation KU Leuven Leuven Belgium; ^5^ Department of Respiratory Diseases University Hospitals Leuven Leuven Belgium; ^6^ Max Planck Research Unit for Neurogenetics Frankfurt am Main Germany

**Keywords:** lung transplantation, NAAT, point‐of‐care test, rapid antigen test, SARS‐CoV‐2

## Abstract

Point‐of‐care tests may play a valuable role in reducing the risk of donor‐derived SARS‐CoV‐2 transmission in lung transplantation.

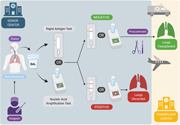

1

Severe acute respiratory syndrome coronavirus 2 (SARS‐CoV‐2) has dramatically affected lung transplant (LTx) programs worldwide. A major challenge in LTx is the risk of donor‐derived viral transmission. Current guidelines advise screening of deceased lung donors with chest computed tomography (CT) and recommend reverse transcription (RT)‐PCR testing for *SARS‐CoV‐2* RNA on a lower respiratory tract (LRT) sample within 72 h before procurement.^1^

There are reports about two cases of donor‐derived SARS‐CoV‐2 transmission during LTx in the literature. Based on a negative NP swab, the lungs were accepted but after LTx, viral infection was detected with RT‐PCR on an LRT sample from the donor.^2^, ^3^ For one recipient, COVID‐19 had a fatal outcome, 60 days after LTx, with a cycle threshold (Ct) value of 8.5, reflecting a high viral load in the LRT sample.^2^ In another case, donor‐derived transmission was prevented by RT‐PCR detection of SARS‐CoV‐2 RNA in an LRT sample obtained at the time of procurement, after a prior negative NP swab.^3^

Viral RNA can persist in the lung for a long time after the acute phase of infection. We reported a double LTx from a donor who was convalescent from mild COVID‐19 (occurring 3 months earlier) and who tested twice negative on NP swab RT‐PCR. No donor‐derived transmission occurred. RT‐PCR on a biopsy of the donor lung before LTx revealed a low viral load with a Ct value of 35, reflecting persistence of viral RNA. Viral culture on the same sample was negative.^4^, ^5^ Interestingly, in samples of the respiratory mucosa of the nasal cavity in the same donor, no persistence of viral RNA was detected.^6^

Several cases of liver, kidney, and heart transplantation with organs from RT‐PCR positive donors have been reported but no donor‐derived SARS‐CoV‐2 transmission has occurred.^7^, ^8^

Despite the observation of SARS‐CoV‐2 RNAemia, no cases of transmission through blood product or stem cell transfusions have been reported.^9^ The viral load detected in blood samples of COVID‐19 patients is typically low (Ct value >30) and virus has not been isolated from blood in cell culture, suggesting that the potential for hematogenous transmission of SARS‐CoV‐2 is low.^9^, ^10^

LTx programs are balancing the risk of donor‐derived transmission with rejecting noninfected, potentially suitable donor lungs. RT‐PCR does not allow to differentiate between persistence of viral RNA, viral shedding, and ongoing viral replication. Access to viral cultures in daily practice is impractical, leaving physicians with the Ct value as an indirect marker for viral load and infectivity. Defining a Ct value as a universal threshold for infectivity is not possible, with laboratories using different protocols and RT‐PCR primer sets (e.g., E, N, or S genes). Based on current evidence, lungs from a donor with a positive RT‐PCR result on an LRT sample are not considered acceptable for transplantation. In case of doubt regarding lung donor infectivity, repeated LRT RT‐PCR testing, careful assessment of the recent history, and judicious chest CT evaluation are indispensable.^1^, ^11^

The incubation period of infection with SARS‐CoV‐2 is highly variable.^12^ Furthermore, the probability of a false‐negative RT‐PCR result decreases gradually from time of exposure/infection to onset of symptoms/high viral load.^13^ Despite absence of symptoms and a negative RT‐PCR during lung donor assessment, a high viral load may have been reached at the time of procurement. Performing RT‐PCR on a NP swab and endotracheal aspirate sample within 24 h before procurement reduces the risk for LTx recipients and healthcare workers.^14^

To further reduce the likelihood of donor‐derived viral transmission and narrow the window of uncertainty between the last RT‐PCR and lung procurement, we here propose the use of point‐of‐care tests (POCTs) on a bronchoalveolar lavage (BAL) sample taken by the procuring surgeon during bronchoscopy. Using POCTs may increase the opportunity of detecting a high viral load and, by extension, replication‐competent virions in the lung grafts. Narrowing the window of uncertainty has gained even more importance with the emergence of the B.1.1.529 (Omicron) variant: its incubation time appears to be shorter.^15^ Therefore, the recommended interval of <72 h between RT‐PCR testing and lung procurement may be too long.^1^

POCTs are easy‐to‐use assays that enable quick, on‐site detection of SARS‐CoV‐2. They include rapid antigen tests (RATs) and various types of nucleic acid amplification tests (NAATs). The performance of POCTs in BAL samples has not been reported except for one NAAT (Bosch Vivalytic), which showed a sensitivity of 96% and specificity of 100%.^16^

RATs are easily transportable, provide a rapid answer after a brief set‐up time, and are user‐friendly (Figure 1). For NP swabs, sensitivity depends on the viral load and type of RAT that is used but specificity is excellent and typically >95%.^17^, ^18^

**Figure 1:**
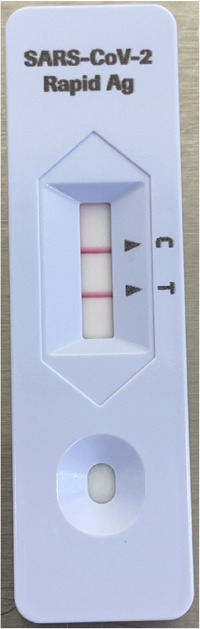
Positive result with a rapid antigen test (RAT) (Roche) on a bronchoalveolar lavage (BAL) sample from SARS‐CoV‐2 positive patient. Cycle threshold (Ct) value of BAL fluid with PCR: in‐house RT‐PCR of Orf1ab (Quantstudio) Ct = 19.5 and rapid RT‐PCR with Roche cobas Liat System Ct = 14.2. PCR, polymerase chain reaction

Several NAATs consisting of a disposable cassette that is inserted in a portable analyzer have been developed for detection of SARS‐CoV‐2 RNA including the Roche cobas Liat System,^19^ Abbott ID NOW,^19^ Mesa Biotech Accula,^20^ Cue Health,^21^ and Lucira Check It.^22^ For NP swabs, sensitivity for these NAATs is higher compared to RATs and specificity >95%.^16^, ^19^, ^20^, ^21^, ^22^ The better performance of NAATs compared to RATs comes with a higher cost and more logistical requirements (transport of analyzer and cassettes, longer set‐up time, more experience required).

The lung procurement team can choose to bring along a RAT or preferably a portable NAAT analyzer to the donor center. After bronchoscopy, the BAL sample is tested and while awaiting the result, macroscopic evaluation of the donor lungs can be performed. When the result of the POCT is positive, the donor lungs are not considered acceptable for LTx.^11^ Figure 2 shows our proposed strategy for the use of the RAT or NAAT assay on a BAL sample obtained at the time of procurement.

**Figure 2:**
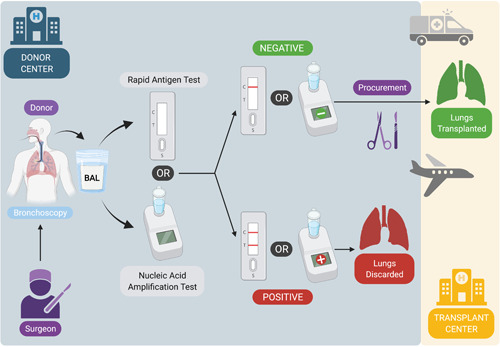
Strategy for the use of a RAT or nucleic acid amplification test (NAAT) to detect SARS‐CoV‐2 infection in BAL fluid sampled from the lung donor at the time of procurement. (Created with BioRender.com)

During the COVID‐19 pandemic, the gap between demand and supply of lung donors has increased. Discarding uninfected, potentially suitable donor lungs must therefore be avoided. For NP swabs, the specificity of RATs and NAATs is >95%.^17^, ^19^, ^20^, ^21^, ^22^ However, to date we do not know the specificity of most POCTs for LRT samples. Only for the Bosch Vivalytic, specificity for LRT samples (100%) has been reported.^16^ Validation of other POCTs for the detection of SARS‐CoV‐2 in LRT samples would increase their usability in the setting of lung donation. This additional safeguard, which is entirely under the control of the procurement team and the transplant center, would help make a timely diagnosis of a SARS‐CoV‐2 infection that was missed due to the window of uncertainty, and would be particularly useful in remote donor regions where RT‐PCR testing may not be available 24/7 and chest CT scans are not available.

We anticipate that the COVID‐19 pandemic will pave the way for a more regular use of POCTs to reduce the risk of donor‐derived transmission of other pathogens that reside in the donor lungs. Timely and on‐site detection of other harmful pathogens such as influenza, aspergillus or mucor could prevent serious morbidity and mortality in LTx recipients.

## CONFLICT OF INTERESTS

The authors of this manuscript have no conflicts of interest to disclose as described by *Immunity, Inflammation and Disease*. The authors confirm that the work described has not been published previously, that it is not under consideration for publication elsewhere, that its publication is approved by all authors and tacitly or explicitly by the responsible authorities where the work was carried out, and that, if accepted, it will not be published elsewhere in the same form in English or in any other language, without the written consent of the copyright holder.

## AUTHOR CONTRIBUTIONS

Conception and design of the work: Jan Van Slambrouck, Peter Mombaerts, Laurens J. Ceulemans. Acquisition, analysis, or interpretation of data: Jan Van Slambrouck, Joost Wauters, Peter Mombaerts, Laurens J. Ceulemans. Assisted in writing: All authors. Approval final draft: All authors.
